# Heart rate changes and myocardial sodium

**DOI:** 10.14814/phy2.15446

**Published:** 2022-09-06

**Authors:** Gabrielle Nelson, Bo Ye, Morgan Schock, Daniel L. Lustgarten, Elisabeth K. Mayhew, Bradley M. Palmer, Markus Meyer

**Affiliations:** ^1^ Department of Medicine Lillehei Heart Institute, University of Minnesota College of Medicine Minneapolis Minnesota USA; ^2^ Department of Medicine and Physiology University of Vermont Larner College of Medicine Burlington Vermont USA

**Keywords:** calcium, heart rate, potassium, sodium

## Abstract

Historic studies with sodium ion (Na^+^) micropipettes and first‐generation fluorescent probes suggested that an increase in heart rate results in higher intracellular Na^+^‐levels. Using a dual fluorescence indicator approach, we simultaneously assessed the dynamic changes in intracellular Na^+^ and calcium (Ca^2+^) with measures of force development in isolated excitable myocardial strip preparations from rat and human left ventricular myocardium at different stimulation rates and modeled the Na^+^‐effects on the sodium‐calcium exchanger (NCX). To gain further insight into the effects of heart rate on intracellular Na^+^‐regulation and sodium/potassium ATPase (NKA) function, Na^+^, and potassium ion (K^+^) levels were assessed in the coronary effluent (CE) of paced human subjects. Increasing the stimulation rate from 60/min to 180/min led to a transient Na^+^‐peak followed by a lower Na^+^‐level, whereas the return to 60/min had the opposite effect leading to a transient Na^+^‐trough followed by a higher Na^+^‐level. The presence of the Na^+^‐peak and trough suggests a delayed regulation of NKA activity in response to changes in heart rate. This was clinically confirmed in the pacing study where CE‐K^+^ levels were raised above steady‐state levels with rapid pacing and reduced after pacing cessation. Despite an initial Na^+^ peak that is due to a delayed increase in NKA activity, an increase in heart rate was associated with lower, and not higher, Na^+^‐levels in the myocardium. The dynamic changes in Na^+^ unveil the adaptive role of NKA to maintain Na^+^ and K^+^‐gradients that preserve membrane potential and cellular Ca^2+^‐hemostasis.

## Graphical abstract

Increasing heart rates lead to a delayed activation of the sodium potassium ATPase as reflected in an initial increase in sodium levels followed by lower levels. The reverse is observed with heart rate lowering.

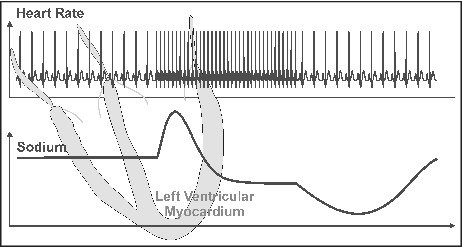

## 1 INTRODUCTION

Intracellular calcium (Ca^2+^) ion levels control cardiomyocyte contraction and relaxation (Ringer, 1883). Triggered by the action potential and facilitated by its concentration gradient, Ca^2+^ enters cardiomyocytes mainly through voltage‐dependent L‐type Ca^2+^‐channels to initiate excitation‐contraction coupling (Ebashi & Endo, 1968). Cumulative Ca^2+^‐entry depends on a variety of factors and conditions. Chief among them is the frequency with which Ca^2+^‐channels open. As a result, higher heart rates allow more Ca^2+^ to enter the cell over a given time interval. The increase in Ca^2+^‐load of intracellular stores, of which the sarcoplasmatic reticulum (SR) is most important, effectively augments contractile force and is a basis of the cardiac force‐frequency relationship (Pieske et al., 1995). To prevent Ca^2+^‐overloading, mechanisms operative to extrude cellular Ca^2+^ have to match this increase in Ca^2+^‐influx as heart rate rises (Eisner et al., 2017). This is accomplished by sarcolemmal Ca^2+^‐extrusion systems, dominated by the Na^+^/Ca^2+^‐exchanger (NCX), whereas the sarcolemmal Ca^2+^‐ATPase only provides a minimal contribution (Bers & Bridge, 1989). The NCX depends on the transmembrane Na^+^‐gradient as its driving force (Reuter & Seitz, 1968). As the cardiac action potential is initiated by Na^+^ entering the cells, higher heart rates will also increase cellular Na^+^‐influx, which, if not counteracted, will reduce the Na^+^‐gradient and thus the ability of the NCX to efficiently extrude Ca^2+^. Up to a certain degree, the accumulation of cellular Ca^2+^ levels will increase contractility, a mechanism that has also been implied to underlie the effects of digitalis glycosides (Blaustein, 1977).

Based on historic studies with Na^+^‐sensing micropipettes and first‐generation fluorescent Na^+^‐indicators, i.e., sodium‐binding benzofuran isophthalate (SBFI), it has been argued that cellular Na^+^‐levels rise and remain high with higher heart rates without consideration of the electrogenic sodium/potassium ATPase (NKA) response to heart rate (Cohen et al., 1982; Donoso et al., 1992; Langer, 1968; Pieske et al., 2002; Thomas, 1972). Tachycardia‐induced Na^+^‐elevations in normal and diseased human myocardium obtained from patients with heart failure with reduced ejection fraction have also been put forward to explain some of the contractile abnormalities observed in heart failure (Pieske et al., 2002). In contrast, when using the fluorescent Na^+^‐probe Asante NaTRIUM Green (ANG‐2), we recently reported that Na^+^‐levels do not appear to substantially change with tachycardia in the control myocardium and diseased myocardium of patients with heart failure with preserved ejection fraction (Runte et al., 2017). Furthermore, total myocardium Na^+^ and potassium (K^+^) levels in unstimulated myocardium were unchanged despite markedly elevated Ca^2+^‐ levels.

Here, we provide qualitative Na^+^‐recordings obtained in rat and human myocardium that reassess the temporal relationship of heart rate changes on Na^+^ with simultaneous assessments of Ca^2+^ and contractile force. In addition, we model the effects of increased heart rates on the membrane potential at increasing sodium levels. Our findings suggest a delayed sodium/potassium ATPase response (NKA) with heart rate changes that were confirmed in the coronary effluent of human subjects after changes in heart rate.

## 2 METHODS

### 2.1 General approach

This study included data obtained in Sprague Dawley rat papillary muscles and human myocardial left ventricular tissue. All procedures were performed in accordance with the Guide for the Care and Use of Laboratory Animals (US Department of Health, Education, and Welfare, Department of Health and Human Services, NIH Publication 85–23), and approved by the University of Vermont and University of Minnesota Animal Care and Use Committees. Sprague Dawley rats were anesthetized and euthanized with isoflurane (5% or higher) and cervical dislocation followed by thoracotomy and harvest of the heart. The hearts were transferred into Tyrode solution at 4°C with 30 mM 2,3‐butanedione monoxime (BDM) and the left ventricular cavity was exposed by an incision along the anterior ventricular septum.

As previously described, the patients were recruited to undergo an intraoperative LV myocardial biopsy from among those scheduled for coronary artery bypass grafting at the University of Vermont Medical Center in Burlington, VT (Runte et al., 2017). All patients provided informed consent for a protocol approved by the University of Vermont Institutional Review Board. Left ventricular anterior wall myocardial biopsies were obtained from the epicardial surface as previously described.

### 2.2 Muscle preparation and parameters

Electrically excitable strip preparations were sculpted from the biopsies and used to assess mechanical parameters and the Ca^2+^ and Na^+^‐handling. Strips of papillary muscle or epicardial myocardium were micro‐dissected along the principal fiber orientation. As previously described, platinum omega‐shaped clips were attached to both ends of the strips and transferred into a fluorescent dye loading chamber (Runte et al., 2017). The fluorescent dye mixture consisted of the acetoxymethyl‐ester form of the calcium dye Fura‐2 (0.05 mmol/L) and sodium dye Asante NaTRIUMGreen‐2 (ANG‐2, 0.05 mmol/L) in 30 mM BDM Krebs solution containing 0.1% pluronic acid and 0.5% dimethyl sulfoxide. ANG‐2 was chosen over SBFI for its greater selectivity for Na^+^ than K^+^ (~41‐fold versus ~18‐fold) and the higher fluorescence quantum yield (0.2 vs. 0.08) that facilitates more selective Na^+^ measurements. (Szmacinski & Lakowicz, 1997)

The strips were loaded in a light‐protected 1.5 ml containment at room temperature for 1–2 h. Thereafter, the strips were transferred to a tissue bath of oxygenated Tyrode solution. A KG4 force transducer (Scientific Instruments) and stimulation unit (MyoPacer; IonOptix, MA) were attached to the Omega clips in the tissue bath. The strip was subject to a 5‐to‐15‐min equilibration period where preload was minimal at a stimulation rate of 0.5 or 1 Hz to allow for the wash‐out of BDM. After isometric force transients were stable, preload was gradually increased until the maximum developed force was reached (Lmax). Force, Ca^2+^, and Na^+^ were simultaneously assessed on the IonWizard digital recording system (version 6.3; Ion Optix, MA) as described (Runte et al., 2017). The principal stimulation rates were 60 and 180/min at physiological temperatures and sufficient oxygenation levels were confirmed by stopping the circulating solution (Meyer et al., 1998). The average cross‐sectional areas were 1.04 ± 0.26 (SD) for the rat preparations and 1.02 ± 0.09 (SD) mm^2^ for the human myocardium.

### 2.3 Sodium and calcium measurements

After the loading period strips were transferred to a tissue bath that was custom‐designed and mounted on an inverted microscope (Nikon ECLIPSE TS100 and a T1‐SM stage). The excitation light source was a 75‐W xenon arc‐bulb (UXL‐75XE; Ushio Corp) and a galvo‐controlled dichroic mirror, which interleaved the excitation bandpass filtered at 380 nm and 485 nm (IonOptix, MA). These filters were selected because Fura‐2 is not excited at 485 nm nor is ANG‐2 excited at 380 nm. Continuous recording of the emitted fluorescence at 510 nm using a PMT400 photomultiplier provided simultaneous monitoring of free intracellular Ca^2+^ by Fura‐2 and intracellular Na^+^ by ANG‐2 at an interleaving frequency of 250 Hz (Runte et al., 2017). Na^+^‐peak and ‐trough recordings with rate changes were analyzed for time to peak (trough) signal and total peak or trough times to return to baseline. The expected reduction in the Na^+^‐signal was confirmed after adding Lidocaine (300 μM) to the circulating solution. When comparing the initial rate‐induced sodium peak before and after it was reduced by 49% ± 9% after Na^+^ channel inhibition with Lidocaine (*n* = 4, *p* < 0.05).

### 2.4 Modeling of NCX Activity at increasing intracellular sodium levels

The equilibrium membrane potentials for Na^+^ and Ca^2+^ were calculated using the Nernst equation:

(1) $$E_{x}=\frac{\mathrm{RT}}{\mathrm{ZF}}\text{In}\left(\frac{{\left[X\right]}_{o}}{{\left[X\right]}_{l}}\right)$$

where *E*_*X*_ = membrane potential preventing any net movement of the *X* ion across a membrane, [*X*]_*o*_ and [*X*]_*i*_ are concentrations of the *X* ion outside and inside the cell membrane, respectively, *R* = universal gas constant (8.314 J.K^−1^.mol^−1^), *T* = absolute temperature (293 K), *z* = number of charges for the ion, and *F* = Faraday's constant (96,485 J.V^−1^.mol^−1^).

Due to the exchange of 3 Na^+^ ions for each Ca^2+^ ion, the equilibrium potential for NCX activity is given as (Bers & Weber, 2002):

(2) $$E_{\mathrm{Na}/\mathrm{Ca}}=3E_{\mathrm{Na}}-2E_{\mathrm{Ca}}$$

Forward NCX activity, i.e., Na^+^ influx coupled with Ca^2+^ efflux, is proportional to the difference between *E*_Na/Ca_ and the membrane potential, *E*_*m*_. When *E*_Na/Ca_ > *E*_*m*_, Ca^2+^ is extruded from the cell. When *E*_Na/Ca_ < *E*_*m*_, NCX is in reverse mode, and Ca^2+^ enters the cell via NCX.

NCX activity and its dependence upon intracellular Na^+^ were calculated based on the constituents of physiological solutions with extracellular Na^+^ ([Na^+^]_*o*_) = 140 mM and Ca^2+^ ([Ca^2+^]_*o*_) = 1.8 mM. Intracellular Na^+^ ([Na^+^]_*i*_) is reported between 10 and 16 mM for rat cardiac myocytes (Despa et al., 2002). For purposes of modeling the effects of varying [Na^+^]_*i*_ on NCX activity, we used values of [Na^+^]_*i*_ = 7.5, 10, 12.5, or 15 mM in the Nernst equation to cover changes in [Na^+^]_*i*_ from 10 mM. For intracellular Ca^2+^, we assumed diastolic [Ca^+^]_*i*,dias_ = 100 nM and systolic [Ca^+^]_*i*,sys_ = 1 μM in accordance with accepted orders of magnitudes for these measures (Ward et al., 2003). Membrane potential, *E*_*m*_, was assumed to be −90 mV during diastole and + 10 mV during systole. For the purposes of estimating NCX activity and its dependency upon intracellular sodium, the duration of systole was assumed to be 100 ms at both 60 bpm and 180 bpm.

### 2.5 Cardiac effluent measurements

As previously reported adult patients in sinus rhythm without hypertension and paroxysmal atrial fibrillation scheduled to undergo elective radiofrequency ablation were enrolled in a University of Vermont IRB‐approved study (Silverman et al., 2020). The present data includes additional measurements obtained in 12 control subjects not previously reported. Normotensive patients with a preserved left ventricular ejection fraction (≥50% by echocardiography), normal wall motion, and end‐diastolic volume index (≤75 ml/m^2^) qualified. Briefly, after the informed consent was obtained, all patients underwent general anesthesia. The right and left femoral veins were cannulated under fluoroscopic guidance with a modified‐Seldinger technique, each with multiple catheters placed including a 7 Fr deflectable electrode catheter, a pacemaker lead, and an 8 Fr AcuNav intracardiac echocardiogram (ICE) catheter. A SL1 sheath was advanced into the coronary sinus for blood sampling. The following blood tests were obtained in sinus rhythm and 15–60 s after starting or stopping pacing at 125 bpm. All samples were drawn into 5 cc heparin‐coated arterial blood gas syringes and placed on ice. Immediately following the measurements, the samples were analyzed for Na^+^ and K^+^‐levels in the clinical pathology laboratory. All catheters are used as part of standard ablation therapy.

### 2.6 Statistical approach

As specified the data are presented as means with standard deviations or medians and interquartile ranges if not otherwise indicated. The coronary effluent data were analyzed with paired Student *t*‐tests. Statistical sensitivity analyses were performed using nonparametric testing to confirm significance. Parametric *p*‐values are reported. Formal tests utilized a 5% significance level. Data analysis was conducted using GraphPad Prism 9.1.2.

## 3 RESULTS

### 3.1 Sodium and rate changes in rat myocardium

**FIGURE 1:**
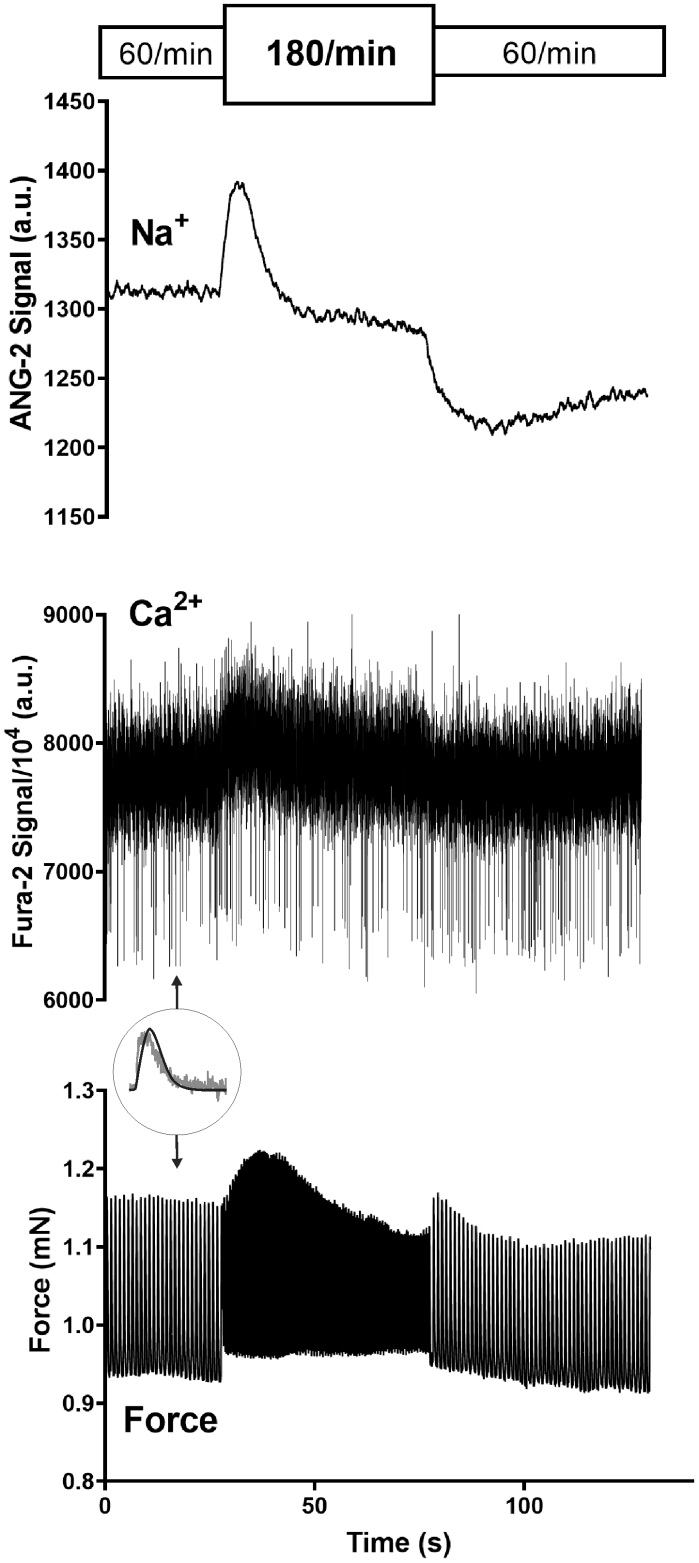
Dynamic changes in intracellular sodium with rate changes. Simultaneous Ca^2+^ (Fura‐2), Na^+^ (ANG‐2), and force tracings in rat myocardium exposed to stimulation rate changes from 60/min to 180/min and back to 60/min. The circular inlay presents a single force (black) and Ca^2+^ transient (gray)

**FIGURE 2:**
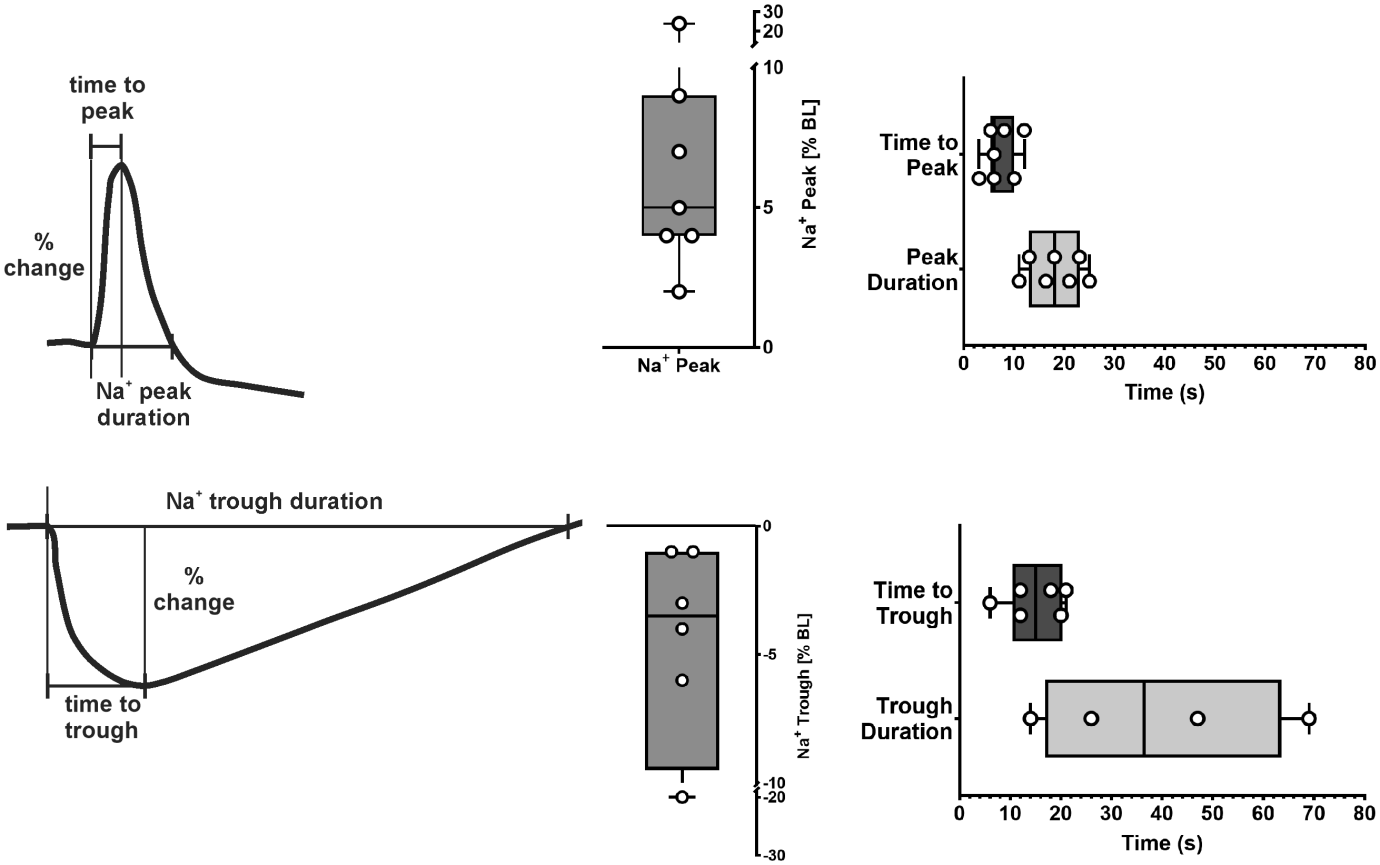
Sodium Peak and Trough Comparison. Schematic presentation of the Na^+^‐peak (top) and the Na^+^‐trough (bottom) with box and whisker plots of the measured magnitude of peak (trough), time to peak, and peak (trough) duration. The data are presented as box and whisker plots that visualize the full range of the data

**FIGURE 3:**
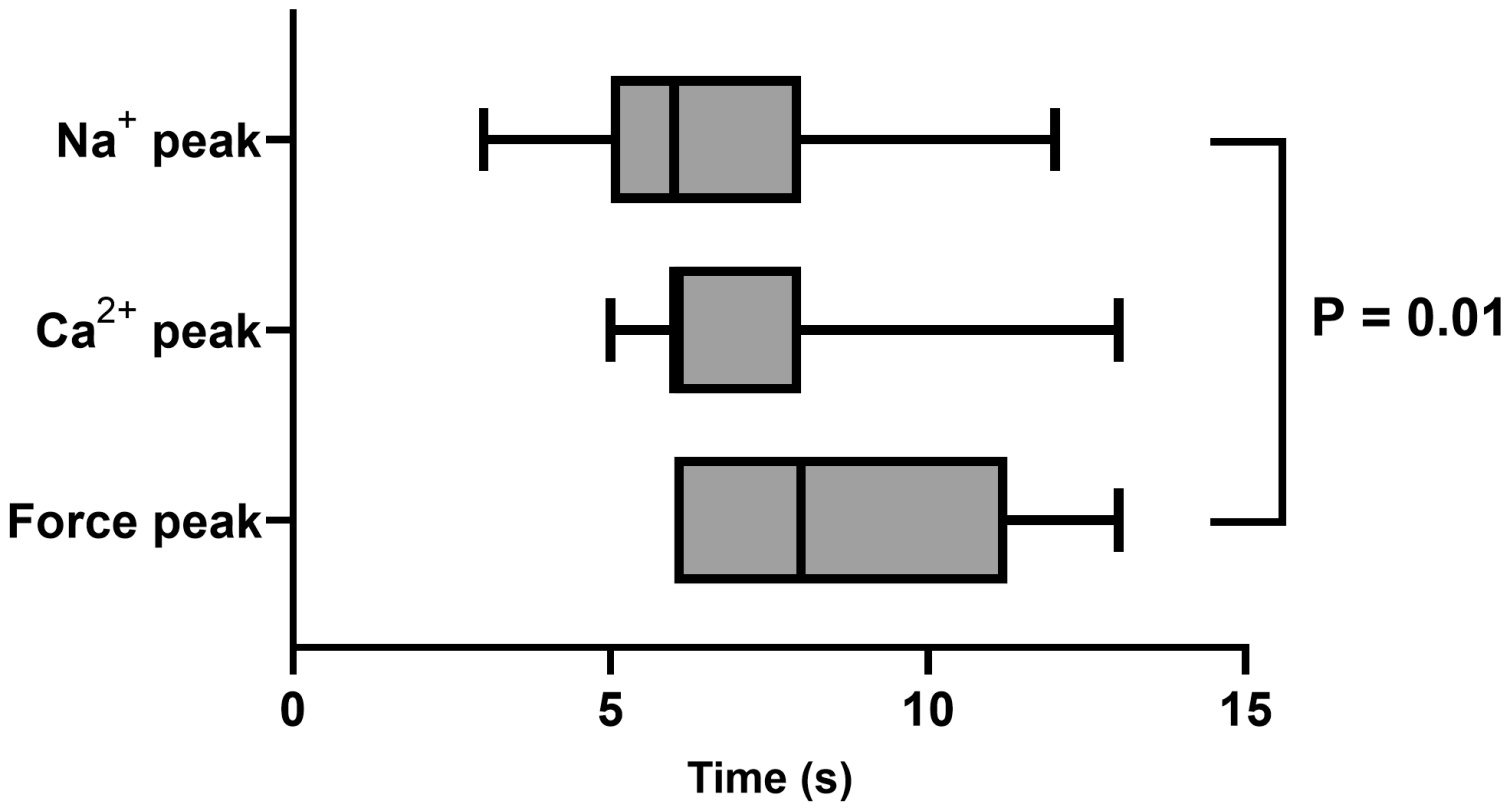
Force, Ca, and Na Time to Peak Comparison. Box and whisker plots of time to peak for Na^+^, Ca^2+^, and systolic force after an increase in the stimulation rate from 60/min to 180/min demonstrating the sequential temporal coupling. When the times to the Na^+^‐peak and the force peak were compared the time to the force peak was significantly delayed

**FIGURE 4:**
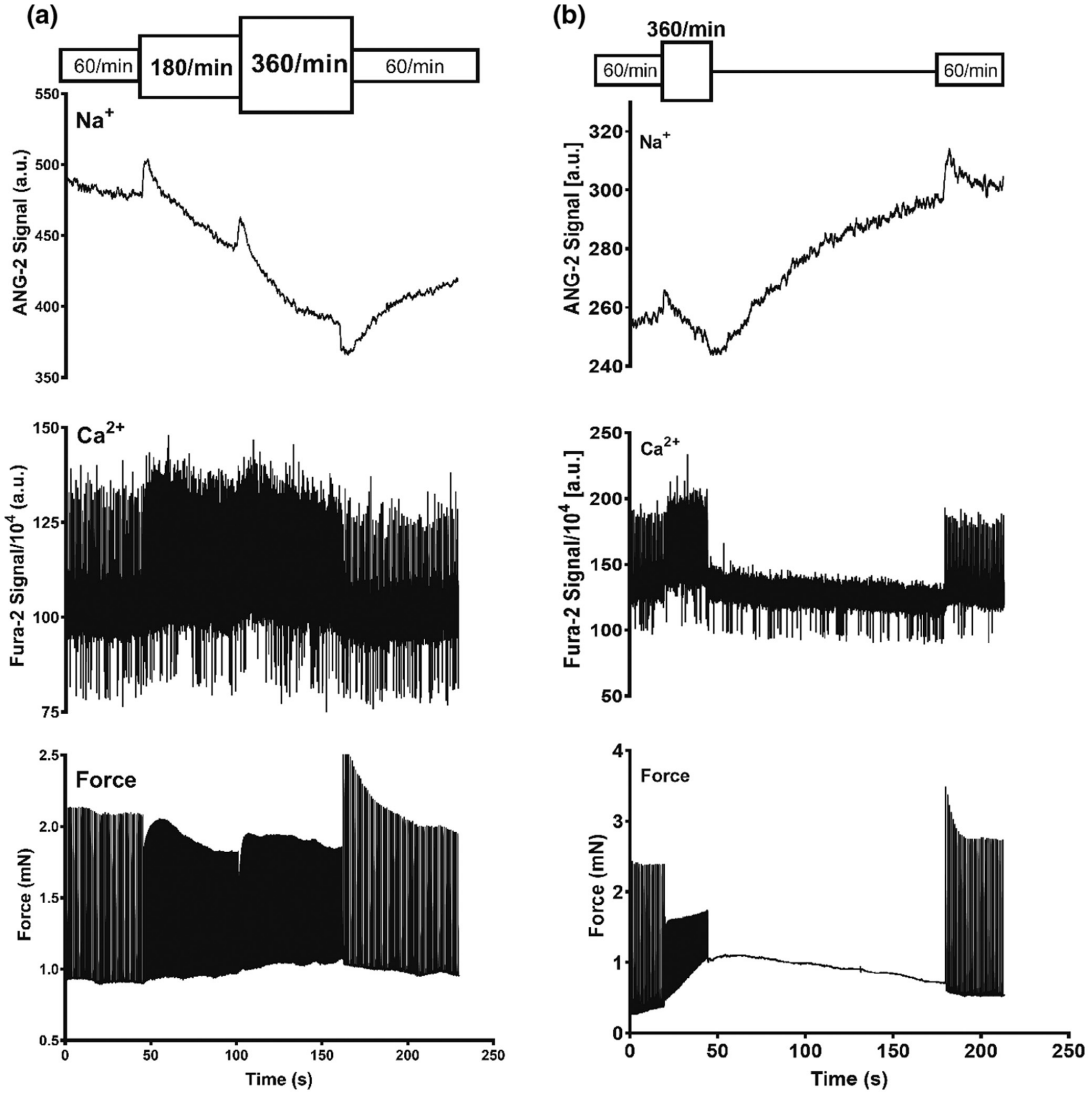
Additional Increase in Stimulation Rate and Stimulation Cessation. Panel (a) demonstrates a Na^+^ and Ca^2+^ and force tracing in response to a stepwise increase in stimulation frequency from 60/min, 180/min, and 360/min demonstrating a second sodium peak and corresponding changes in Ca^2+^ and force. Panel (b) demonstrates the restoration of higher Na^+^‐levels after stimulation cessation to baseline levels followed by pacing at 60/min

An increase in stimulation rate from 60/min to 180/min was followed by an immediate rise in cellular Na^+^ as shown in Figure 1. After reaching a peak, Na^+^ generally dropped to lower levels. When the rate was then returned from 180/min to 60/min the Na^+^‐signal initially dropped and formed a trough. Thereafter Na^+^ rose. Corresponding changes in the systolic and diastolic Ca^2+^‐levels were observed. The transient Na^+^‐peak was closely followed by a transient peak in force development. As summarized in Figure 2 the time to peak Na^+^ with tachycardia was significantly faster than the time to trough with a rate reduction to suggest differential regulation (in sec: 7.2 ± 1.1 vs. 14.8 ± 2.4, *p* = 0.028). Correspondingly, the magnitude of the Na^+^‐peak was more pronounced than the Na^+^‐trough (%baseline: 8 ± 3 vs. 6 ± 3, *p* = 0.016). Figure 3 summarizes the lag time of the Na^+^, Ca^2+^, and developed force peaks after increasing the rate from 60/min to 180/min demonstrating the temporal relationship. In all experiments, Na^+^ peaked first. As shown in Figure 4 an additional increase in stimulation rate from 180/min to 360/min resulted in the same pattern of the Na^+^‐signal whereas a cessation of stimulation after an initial drop in Na^+^ levels was followed by partial or full Na^+^ recoveries over prolonged time intervals.

### 3.2 Sodium and rate changes in isolated human myocardium

**FIGURE 5:**
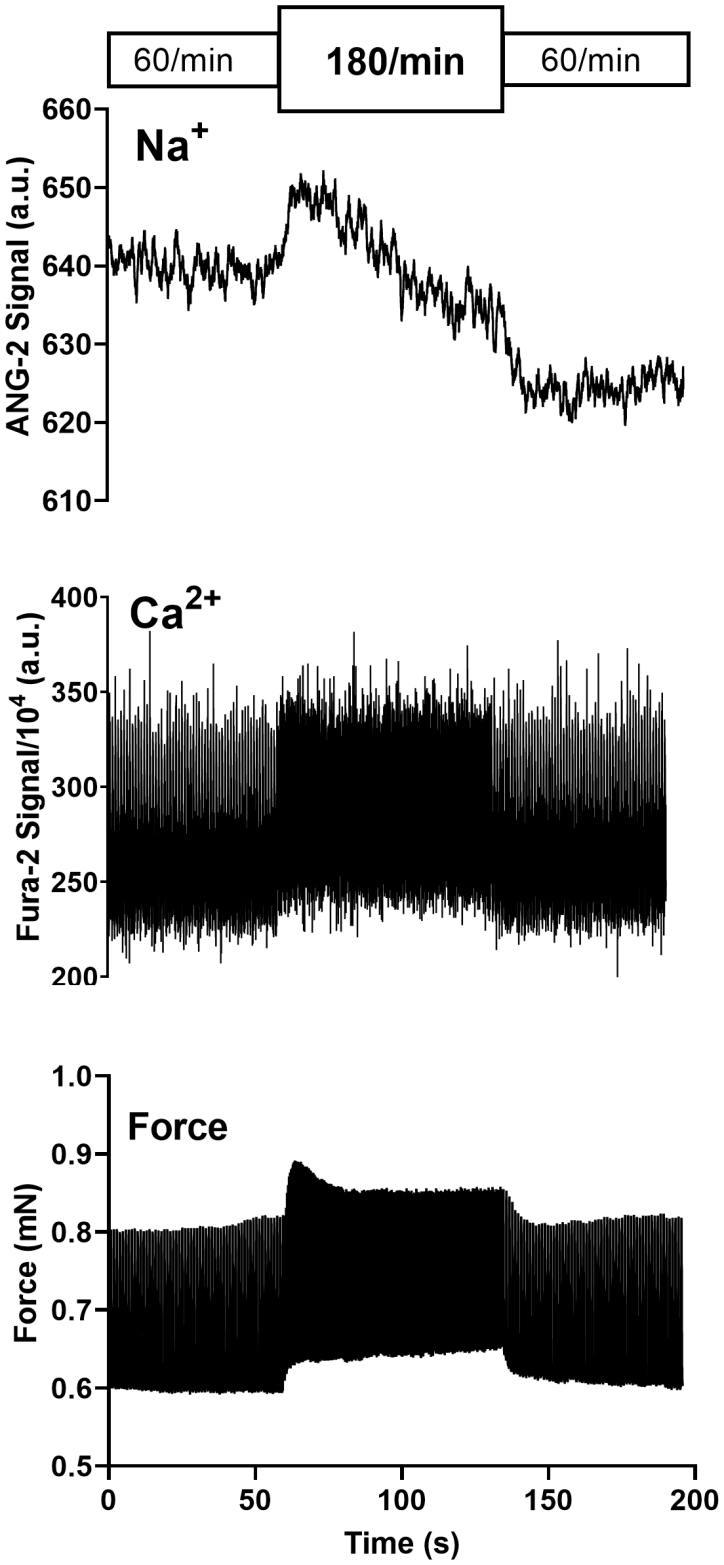
Human Example. Simultaneous Na^+^ (ANG‐2), Ca^2+^ (Fura‐2), and force tracings in human myocardium obtained from patients with coronary artery disease and hypertension with rate changes from 60/min to 180/min and back to 60/min showing a similar but less pronounced pattern compared to rat myocardium

As shown in Figure 5, we were able to document similar but somewhat less pronounced tachycardia‐induced changes in Na^+^, Ca^2+^, and force in three human preparations obtained from patients with hypertensive heart disease to reveal a Na^+^‐peak followed by lower Na^+^‐levels. The most likely explanation for this observation is a delayed increase in sodium/potassium ATPase (NKA) activity, whereas a delayed slowing of the NKA activity would explain the Na^+^ trough after a decrease in stimulation rate. The delay in the adjustment of NKA activity after rate changes were confirmed in vivo by measuring potassium (K^+^) and Na^+^ levels in the coronary effluent of control patients.

### 3.3 Coronary effluent

**FIGURE 6:**
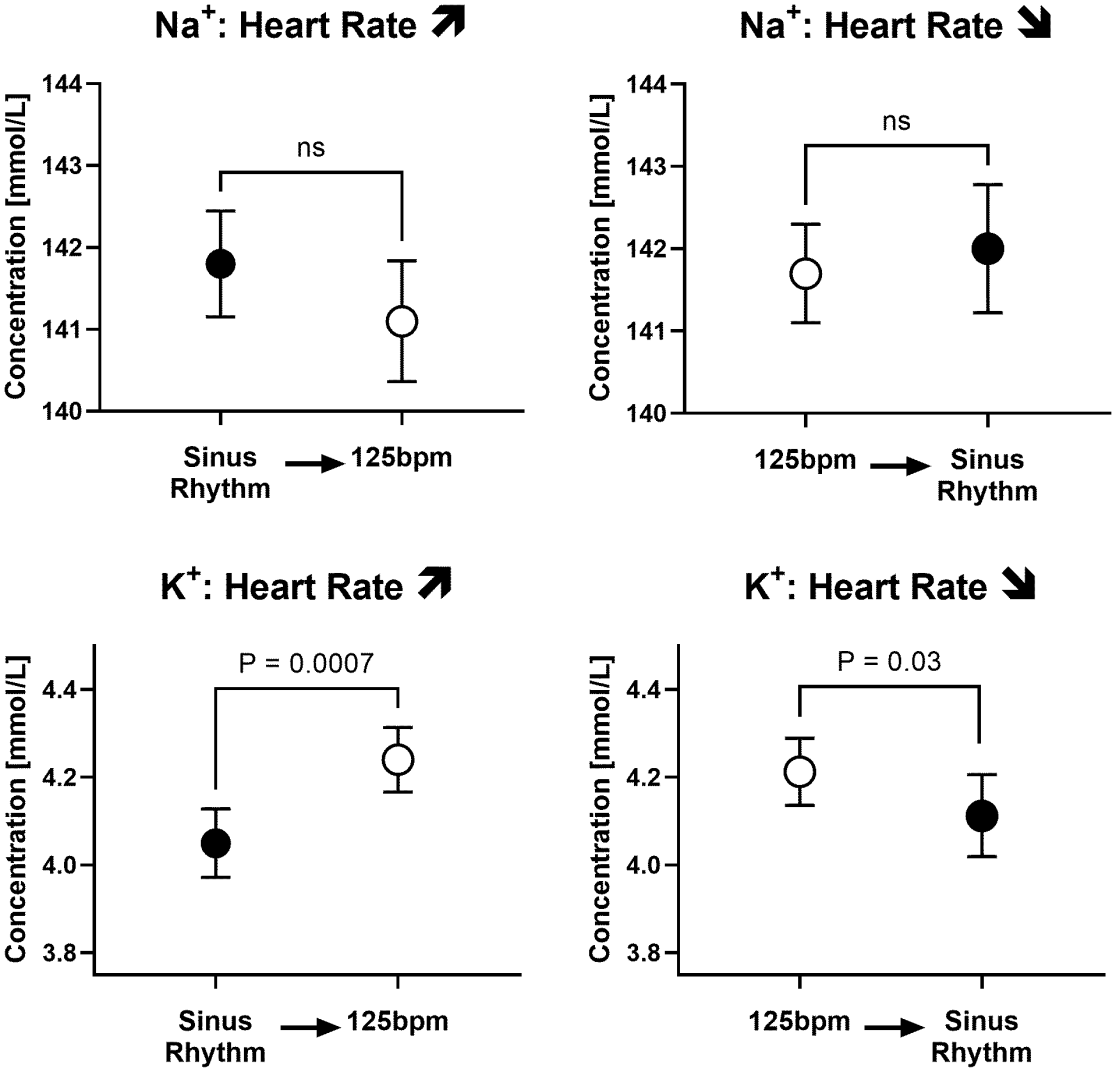
Coronary Effluent. Coronary sinus Na^+^‐ and K^+^‐levels with sinus rhythm and with pacing at 125/min. The samples were drawn at a steady state and 15 to 60 s after changing the heart rate. *p*‐values from paired t‐tests are provided. Error bars are +/− SEM

The baseline characteristics of the studied patients have been reported previously. (Silverman et al., 2020) After an increase in heart rate to 125 bpm coronary sinus K^+^‐ levels rose from 4.05 ± 0.25 at 60 bpm to 4.24 ± 0.23 at 125 bpm (p = 0.0007) to suggest that NKA activity did not yet reach the steady state to compensate for the increase in the repolarizing K^+^‐efflux. Na^+^ levels showed a trend toward lower levels as shown in Figure 6. When the pacing at 125 bpm was stopped, the reverse was observed to again suggest a lag in NKA regulation that results in myocardial K^+^‐retention.

### 3.4 Estimated NCX activity

While we have not quantified intracellular sodium content, the monotonic relationship between ANG‐2 and [*Na* ^+^]_*i*_ permits inferring that [Na^+^]_*i*_ is transiently elevated when the stimulation rate is increased and transiently depressed when the stimulation rate is reduced. The effects of these transient changes on NCX function were estimated as the electrochemical potential that drives NCX activity as described in Equations 1 and 2.

**FIGURE 7:**
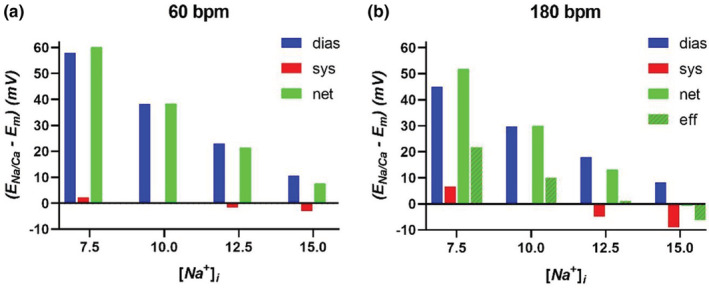
NCX dependence on intracellular Na^+^ and stimulation rate. Effects of changes in intracellular Na^+^ on NCX activity denoted as E_Na/Ca_–E_m_. (a) At 60/min, NCX activities in both diastole and systole are reduced with increasing [Na^+^]i due to the reduction in E_Na_ and the resulting reduction in E_Na/Ca_. At concentrations higher than 10 mM, NCX activity is reversed during systole because of E_Na/Ca_ < E_m_. (b) At 180/min, NCX activity is again reduced with increasing [Na^+^]_i_. However, due to systole now making up a higher fraction of the cardiac cycle, net NCX exchange over a full cycle is more sensitive to the effects of [Na^+^]_i_ compared to 60 bpm. Considering that Ca^2+^ influx is three times higher at 180/min compared to 60/min, the effective (eff) NCX activity is nearly zero at 12.5 mM and negatively valued at 15 mM

Figure 7 illustrates the expected NCX activity for a variety of [Na^+^]_*i*_ values that would be physiologically relevant. Under basal conditions at 60 bpm (Figure 7a), we expect [Na^+^]_*i*_ to be near 10 mM, which is within the currently accepted range of 10–16 mM [Na^+^]_*I*_ for rat cardiac myocytes (Aksentijevic et al., 2018; Despa et al., 2002). At this concentration, *E*_Na_ = 66.6 mV corresponds to the membrane voltage that prevents net inward diffusion of Na^+^ across the membrane. A membrane potential less than 66.6 mV would permit Na^+^ diffusion into the cytosol, albeit very small given the high resistance to Na^+^ movement across the sarcolemma.

During diastole, [*Ca* ^2+^]_*i*_ is 100 nM, and *E*_*Ca*_ = 123.7 mV. Again, Ca^2+^ would diffuse across the membrane into the cytosol when the membrane potential is less than 123.7 mV. And again, this diffusion is very small. The movement of Na^+^ and Ca^2+^ across the sarcolemma is facilitated by NCX with an *E*_Na/Ca_ of −47.5 mV, meaning that 3 Na^+^ ions will move inward for every 1 Ca^2+^ ion moving outward when the membrane potential is less than −47.5 mV. In diastole, the membrane potential (*E*_*m*_) is −90 mV. Therefore, the NCX activity, calculated as (*E*_Na/Ca_–*E*_*m*_) is positive with a value of 38.3 mV (see Figure 7a for [Na^+^]_*i*_ = 10 mM during diastole) and, therefore, NCX extrudes Ca^2+^ from the cell.

During systole at 60 bpm and [Na^+^]_*i*_ = 10 mM, [Ca^2+^]_*i*_ is 1 μM, E_Ca_ = 94.6 mV, and E_NCX_ = 10.7 mV. Because the action potential has plateaued at +10 mV during systole, *E*_Na/Ca_ is similar to *E*_*m*_, and NCX activity is negligible (see Figure 7a for [Na^+^]_*i*_ = 10 mM during systole). The net activity of NCX over a full cardiac cycle, therefore, occurs in diastole alone.

If [Na^+^]_*i*_ were reduced to 7.5 mM, NCX activity during both diastole and systole would be elevated compared to [Na^+^]_*i*_ at 10 mM. If [Na^+^]_*i*_ were elevated to 12.5 or 15 mM, NCX activity during both diastole and systole would be reduced. During systole, however, *E*_Na/Ca_ is less than *E*_*m*_ during the plateau of the action potential, and NCX activity is negatively valued indicating that Ca^2+^ is brought into the cytosol during this portion of the cardiac cycle.

At 180 bpm (Figure 7b), NCX activity is again reduced with elevated [Na^+^]_*i*_, but the net effect is more pronounced at the higher heart rate due to the shorter time period in diastole compared to 60 bpm. With three times the influx rate of Ca^2+^ at 180 bpm and with Ca^2+^ being extruded only during diastole, the effective (eff) NCX activity at 180 bpm can be directly compared with the net NCX activity at 60 bpm. The effective NCX activity is nearly zero at 12.5 mM and negatively valued at 15 mM, highlighting the adverse effects of intracellular Na^+^ elevations on NCX‐mediated Ca^2+^‐extrusion, especially at higher heart rates.

## 4 DISCUSSION

We studied the effects of stimulation rate changes on cellular sodium (Na^+^) and calcium (Ca^2+^) and force development in isolated rats and human myocardium. We also assessed changes in Na^+^ and potassium (K^+^) levels in the coronary effluent of patients after changes in heart rate. The following observations were made:

1. In isolated myocardium, a sudden rise in stimulation rate was associated with a short transient cellular Na^+^‐peak that is followed by a decrease in Na^+^‐levels. The reverse was observed with heart rate lowering resulting in a prolonged Na^+^‐trough followed by an at least partial recovery of Na^+^‐levels.
2. These changes in Na^+^ in isolated myocardium were followed by associated changes in Ca^2+^ and force development that can be explained by the Na^+^‐dependence of the sarcolemmal Na^+^/Ca^2+^ exchanger (NCX).
3. In patients, the dynamic changes in Na^+^ are likely due to delayed activation of the sodium/potassium ATPase (NKA) as evident in K^+^‐levels measured in the coronary venous effluent after changes in heart rate.
4. Multivariate membrane‐potential modeling of the effects of heart rate at different cellular Na^+^‐levels confirmed that increased cytosolic Na^+^‐levels at higher rates would be unfavorable for NCX function.

### 4.1 Myocardial sodium and changes in heart rate

Using a second‐generation Na^+^‐indicator we were able to provide novel qualitative insights into the time course of cellular Na^+^‐hemostasis after heart rate changes. When the stimulation rate was increased from 60/min to 180/min, intercellular Na^+^ rose likely due to an increased rate of Na^+^‐channel openings that enhances cellular Na^+^‐influx. The rise in Na^+^ peaked and then returned to and fell below the steady state Na^+^‐levels at 60/min. Because the sodium/potassium ATPase (NKA) is the only known cellular transporter able to lower Na^+^‐levels, this biphasic time course is most likely due to a delayed but necessary upregulation in NKA activity to preserve the transsarcolemmal Na^+^ and K^+^‐gradients.

The cellular Na^+^/Ca^2+^‐exchanger (NCX) is the main Ca^2+^‐extrusion system that depends on NKA to preserve a Na^+^‐gradient to efficiently remove cellular Ca^2+^ against its own concentration gradient. Higher heart rates not only lead to greater Na^+^‐influx but also greater Ca^+^‐entry through more frequent openings of the voltage‐sensitive L‐type calcium channels. The transient increase in cellular Na^+^‐levels appears to reduce the ability of NCX to sufficiently compensate for the added influx of Ca^2+^ and explains the rise in systolic and diastolic Ca^2+^ levels when heart rate is increased. The elevated Ca^2+^ was closely linked to corresponding changes in force. This putative mechanism would also explain the transient increase in force development that is frequently seen when stimulation rates are increased. The only other known cellular extrusion system for Ca^2+^, the sarcolemmal Ca^2+^‐ATPase, is not able to compensate, most likely due to its limited capacity of less than 1% of total cellular Ca^2+^‐extrusion (Bers & Bridge, 1989).

The explanation for the subsequent reduction in Na^+^‐levels is a higher NKA activity that is first catching up to preserve and then enhance the Na^+^‐gradient, an effect established with skeletal muscle activity (Clausen, 2003). An increase in NKA activity with a higher heart rate is also essential to compensate for the rate‐induced net cellular K^+^‐efflux that restores the membrane potential. If NKA would not compensate for the estimated threefold increase in cellular Na^+^‐influx and K^+^‐efflux when the rate is increased from 60/min to 180/min, the transmembrane concentration gradients for these ions would be gradually lost resulting in a breakdown of the membrane potential. Our studies suggest that the Na^+^‐levels at higher heart rates ultimately equilibrate to lower levels to putatively maintain a higher Na^+^ gradient that facilitates increased NCX‐mediated Ca^2+^‐efflux. That NCX‐mediated Ca^2+^‐extrusion is enhanced at higher rates was also suggested in studies where sarcoplasmatic reticulum (SR) function was completely inhibited. Relaxation times, a surrogate for NCX function, continued to accelerate with increases in stimulation rates (Schwinger et al., 1997). We observed the same in SR inhibition experiments with cyclopiazonic acid and ryanodine in human preparations (Selby et al., 2011). Our understanding of the regulation of cardiac NKA is limited. Besides a direct Na^+^ regulation of cytoplasmic NKA, it is very likely that other mechanisms, e.g., phosphorylation of the NKA or regulatory proteins, e.g., phospholemman, will play a substantial role as described in skeletal muscle. (Clausen, 2003; Fuller et al., 2013; Pirkmajer & Chibalin, 2016)

Similarly, our experiments may offer a more nuanced view of Na^+^‐levels in response to the lowering in stimulation rates. A rate change from 180/min to 60/min is accompanied by a precipitous reduction in Na^+^‐levels that can be explained by a continually high NKA activity that then slows to explain the formation of a Na^+^‐trough before the levels slowly revert to higher Na^+^‐levels. Interestingly, the slowing of NKA is protracted when compared to the acceleration to suggest higher order regulatory mechanisms, which have been discussed elsewhere, e.g. kinases versus phosphatases (Clausen, 2003; Pirkmajer & Chibalin, 2016).

Our findings challenge the widely held view that increases in heart rate are associated with higher cellular Na^+^‐levels. This conceptual framework has been used to explain cellular Ca^2+^‐retention with tachycardia. However, this explanation does not account for the gradient and membrane potential preserving role of NKA which response to changes in demand. To assess if the proposed lag in NKA adaptation with heart rate changes can be directly confirmed in vivo we sampled K^+^ and Na^+^‐levels in the coronary effluent. Historical studies using micropipettes have likely been complicated by an inability to completely seal the membrane in moving cells, a reason why Purkinje cells were used. This would also explain why some historical tracings also documented a post‐stimulation Na^+^ trough (Boyett et al., 1987; Levi & Boyett, 1991). Measurements with the first generation ratiometric Na^+^ indicator SBFI using two excitation wavelengths are complicated by low Na^+^ selectivity and the recent finding that the emission at lower excitation wavelength is insensitive to changes in sodium and tends to invert the ratio, whereas the emission at higher excitation wavelengths directly parallels changes in Na^+^ similar to ANG‐2. (Despa et al., 2004; Frampton et al., 1991). It is noteworthy that the changes in K^+^ in the coronary effluent with rate changes reflect the K^+^ changes reported in historical measurements in the myocardial extracellular space after heart rate changes (Kunze, 1977).

### 4.2 Lag in sodium/potassium ATPase response after heart rate changes

The coronary effluent data obtained in control subjects revealed that an increase in heart rate to 125 bpm followed by a reversal to sinus rhythm is associated with a lag in NKA functional adaptation reflected in a K^+^‐disequilibrium. Whereas the early changes in the K^+^ and Na^+^ coronary effluent suggest K^+^‐efflux from the myocardium with higher heart rate and a trend toward Na^+^‐retention, the reversal of the heart rate from 125 bpm to sinus rhythm suggests a delayed slowing of the higher NKA activity resulting in lower K^+^‐levels in the coronary effluent. It is not surprising that changes in K^+^‐levels are easier to detect because plasma levels are low compared to intracellular levels. A lag in the NKA‐response toward heart rate changes has been hypothesized by Woodbury in 1963 and Langer in 1968 but to our knowledge has never been confirmed in the myocardium (Langer, 1968; Woodbury JW., 1963). We believe that in aggregate our findings confirm the Na^+^‐lag hypothesis, a predicted outcome that was not consistently seen in SBFI or micropipette measurements as discussed.

### 4.3 Thermodynamic considerations

Contrary to the common assumption that higher heart rates are associated with an increase in intracellular Na^+^‐levels, our observation of a brief increase in Na^+^ followed by a decrease is more aligned with thermodynamic requirements for NCX function and the essential role of the NKA. As revealed in our analysis of membrane potentials an increase in intracellular Na^+^‐levels would reduce the ability of NCX to compensate for the increase in Ca^2+^‐influx with higher heart rates. Maintenance of the Na^+^‐ and K^+^‐gradients is the essential role of NKA. This transporter is metabolically protected and under normal circumstances only fails when backup glycolytic ATP production ceases, e.g., with prolonged severe ischemia. Analogous to skeletal muscle, it is a fundamental necessity that an increase in heart muscle activity increases NKA activity (Clausen, 2003).

## 5 LIMITATIONS

NKA activity was not directly measured, but in most circumstances provides the only reasonable explanation for our observations. Although we did not quantify cellular Na^+^‐levels we have internal baseline comparisons that allow for a descriptive analysis of a biphasic response that is tightly associated with predicted changes in Ca^2+^‐handling and force development.

## 6 OVERALL SIGNIFICANCE

In summary, our data suggest that the NKA maintains and potentially optimizes myocardial Na^+^‐levels with heart rate changes. The adjustment of NKA activity with a heart rate increase appears to be faster than with a decrease in heart rate. Because the sarcolemmal Na^+^/Ca^2+^ activity is dependent on the Na^+^‐gradient and membrane potential the secondary effects on Ca^2+^ and force development are most likely explained by the changes in Na^+^.

## AUTHOR CONTRIBUTIONS

All authors contributed and approved the manuscript. GN, BY, MS: rat myocardial studies and manuscript. DL, EM: human studies and manuscript. BP, MM: experimental design, modeling, and manuscript.

## FUNDING INFORMATION

M. Meyer was supported by the Engdahl Family Foundation and a National Institutes of Health Grant (R01 HL‐122744).

## CONFLICT OF INTEREST

The authors report no conflicts of interest regarding this study.
